# Prevalence and impact of myocardial injury among patients hospitalized with COVID-19

**DOI:** 10.3389/fcvm.2023.1202332

**Published:** 2023-08-01

**Authors:** Vu Hoang Vu, Thanh Cong Nguyen, Quang Dang Duy Pham, Dan Ngoc Pham, Le Bao Le, Khoi Minh Le

**Affiliations:** ^1^ Faculty of Medicine, University of Medicine and Pharmacy at Ho Chi Minh City, Ho Chi Minh City, Vietnam; ^2^ Interventional Cardiology Department, University Medical Center Ho Chi Minh City, Ho Chi Minh City, Vietnam; ^3^ Department of Cardiology, University Medical Center Ho Chi Minh City, Ho Chi Minh City, Vietnam; ^4^ Rheumatology Department, University Medical Center Ho Chi Minh City, Ho Chi Minh City, Vietnam; ^5^ Cardiac Imaging Unit, University Medical Center Ho Chi Minh City, Ho Chi Minh City, Vietnam

**Keywords:** COVID-19, SARS-CoV-2, myocardial injury, cardiac injury, troponin, prognosis

## Abstract

**Background:**

Myocardial injury is a prevalent complication observed in patients hospitalized with COVID-19 and is strongly associated with severe illness and in-hospital mortality. However, the long-term consequences of myocardial injury on clinical outcomes remain poorly understood. This study aimed to assess the impact of myocardial injury on both acute-phase and long-term prognosis in COVID-19 patients.

**Methods:**

A retrospective, observational study was conducted on all patients who received treatment at the Intensive Care Center for COVID-19 patient, University Medical Center Ho Chi Minh City (UCICC), from August 3rd, 2021, to October 28th, 2021.

**Results:**

A total of 582 patients were enrolled in the study, of which 55.3% were female. The mean age of participants was 63.3 ± 16.2. Out of these patients, 330 cases (56.8%) showed myocardial injury. Compared to patients without myocardial injury, those with myocardial injury were older and had a higher incidence of chronic diseases including hypertension, ischemic heart disease, atrial fibrillation, heart failure, diabetes mellitus, chronic kidney disease. They also presented with more severe respiratory failure upon admission and showed a more pronounced abnormality in inflammation and kidney function tests. Furthermore, the in-hospital mortality rate was significantly higher in the group with myocardial injury (49.7% vs 14.3%, *p* < 0.001). After adjusting for age, gender, comorbidities, renal function, and disease severity at admission, myocardial injury emerged as an independent risk factor for in-hospital mortality (OR = 3.758, 95% CI 1.854–7.678, *p* < 0.001). Among successfully discharged COVID-19 patients, the all-cause mortality rate after a median follow-up of 18.4 months was 7.9%. Patients with myocardial injury had a significantly higher long-term mortality rate compared to those without myocardial injury (14.0% vs. 3.2%, *p* < 0.001). However, multivariable Cox regression analysis did not find myocardial injury to be a significant predictor of long-term mortality (HR = 2.128, 95% CI 0.792–5.712, *p* = 0.134).

**Conclusions:**

Myocardial injury is a common and serious complication in hospitalized COVID-19 patients, associated with increased in-hospital mortality. However, it does not significantly impact long-term mortality in successfully discharged COVID-19 patients.

## Introduction

The COVID-19 (Coronavirus Disease 2019) pandemic, caused by the SARS-CoV-2 (Severe acute respiratory syndrome coronavirus 2) and its variants, has had a profound global impact. As of March 2023, the total number of infections worldwide has surpassed 760 million and continues to rise. The COVID-19 pandemic has caused the deaths of over 6.8 million people, resulting in significant economic and social disruption (source: World Health Organization). While the respiratory system is primarily affected, emerging evidence suggests that SARS-CoV-2 also wreaks havoc on the cardiovascular system, leading to diverse manifestations such as myocardial injury, myocardial infarction, arrhythmias, heart failure, thromboembolism, and cerebrovascular events (1–4). Notably, myocardial injury, characterized by elevated serum troponin levels, has garnered considerable attention, occurring in 7%–36% of hospitalized COVID-19 patients (1, 5–7). Angiotensin-converting enzyme 2 (ACE2) receptors, which are abundant in cardiac muscle cells, serve as the gateway for the virus to invade the cell. Respiratory failure, hypoxemia, hemodynamic disorders, hypercoagulability, infection, cytokine storm, and other immune responses also contribute to myocardial injury (1–3, 8).

Numerous studies have demonstrated that myocardial injury is a common complication associated with critical condition and death in SARS-CoV-2-infected patients (6, 9). However, the long-term prognostic significance of myocardial injury after hospital discharge remains unclear due to limitations in sample sizes and follow-up durations in existing studies (10–12). Recent evidence indicates that SAR-CoV2 induces persistent systemic inflammation and organ injury, which can have prolonged effects lasting for months (13). Hence, it is imperative to ascertain the precise influence of myocardial injury on clinical outcomes to facilitate the development of effective management strategies, optimize resource allocation, and minimize costs. To address this critical knowledge gap, our study aims to assess the prevalence of myocardial injury, identify prognostic factors for in-hospital mortality, and most importantly, evaluate the impact of myocardial injury on long-term mortality in hospitalized COVID-19 patients. The results of this study can provide crucial insights into the diagnosis, treatment, and monitoring of this complication, potentially leading to better patient outcomes.

## Materials and method

### Study design and participants

The study was conducted at the Intensive Care Center for COVID-19 patient, a specialized unit within University Medical Center Ho Chi Minh City (UCICC), Vietnam. Our investigation included all adult patients aged 18 years or above who received treatment for COVID-19 between August 3, 2021, and October 28, 2021. The diagnosis and grading criteria were based on the Guidelines for Diagnosis and Treatment of COVID-19 issued by the Ministry of Health of Vietnam. A definitive diagnosis of SARS-CoV-2 infection was established based on the positive test results obtained through real-time polymerase chain reaction (RT-PCR). Detailed definitions of severity grades for COVID-19 patients can be found in Supplementary Methods S1. Patients with moderate or higher COVID-19 infection or those with underlying medical conditions indicated for hospitalization. As for UCICC, priority was given to the admission of severe and critical cases. The inclusion criteria consisted of patients who underwent at least one high-sensitive cardiac troponin (hs-cTn) test within 48 h of admission. The selection between high-sensitive troponin T (hs-TnT) or high-sensitive troponin I (hs-TnI) was made randomly, depending on the availability of the laboratory, and the reference values provided by the manufacturer were utilized. The highest value of troponin in the collected samples was used. Patients who were diagnosed with acute coronary syndromes were excluded from the study (Supplementary Methods S2). Sample size calculation was not performed, and all patients meeting the inclusion criteria were included in the study.

### Data collection

The clinical data, diagnostic information, treatment details, and in-hospital outcomes of patients were obtained from electronic medical (EMR) records used by UCICC. Demographic characteristics such as age and sex were collected. The presence of cardiovascular risk factors and diseases, as well as other relevant diagnoses including hypertension, coronary artery disease, atrial fibrillation, heart failure, diabetes, chronic kidney disease, chronic lung disease, and cancer, were identified through the patient's medical history. Chronic kidney disease was defined as a persistent abnormality in renal function with a glomerular filtration rate <60 ml/min/1.73 m^2^. Admission laboratory investigations included full blood count, biochemical analysis, liver and renal function, inflammatory markers (C reactive protein (CRP) and procalcitonin (PCT)), interleukin 6 (IL-6), D-Dimer and cardiac biomarkers (hs-TnT or hs-TnI), as well as N-terminal-proB-type natriuretic peptide (NT-proBNP). Patients with COVID-19 were divided into two groups based on the presence or absence of myocardial injury, which was defined as an elevation of cardiac enzyme values above the 99th percentile of the reference upper limit (14 ng/L), irrespective of electrocardiogram abnormality and echocardiography findings (14).

### Follow-up and study endpoint

The discharge criteria for COVID-19 patients included a reduction in clinical symptoms, absence of fever for at least 3 days, and negative results on RT-PCR testing for SARS-CoV-2 or a cycle threshold value ≥30 (specific gene not required), or a negative result on a rapid antigen test for SARS-CoV-2. The survival of all patients successfully discharged was monitored through telephone interviews or clinical examinations at the outpatient clinic for up to a maximum of 20 months or until the occurrence of the primary outcome. The primary outcome recorded in this study was death from any cause.

### Ethical considerations

The study did not affect the process of the patient's diagnosis, treatment and follow-up and approved by the local ethics committee of University Medical Center Ho Chi Minh City, number 04/HĐĐĐ-ĐHYD. The statement also indicates that the patient or their representative did not sign a consent form to participate in the study due to the outbreak of the pandemic.

### Statistical analysis

The data were analyzed using the Statistical Package for Social Sciences (SPSS) software version 22.0. We used frequency and percentages as descriptive measures for qualitative variables. Proportions were compared using either the chi–squared test or Fisher's exact test. For quantitative variables, normal distribution was assessed using the Shapiro-Wilk test. Variables with normal distribution were reported as mean ± standard deviation, while variables with non-normal distribution were reported as median. To compare the mean and median of two groups, *t*-test and Mann–Whitney *U* test were used, respectively. In order to perform regression analysis, variables such as NT-proBNP, BNP, interleukin-6, ferritin, and D-Dimer were logarithmically transformed.

We conducted a multivariable logistic regression model to examine the association between myocardial injury and in-hospital mortality in COVID-19 patients. Model 1 was adjusted for demographic factors including age and sex. Model 2 incorporated the variables from model 1 and comorbidities such as hypertension, coronary artery disease, atrial fibrillation, heart failure, diabetes and chronic kidney disease. Adjusted model 3 included the variables from model 2 as well as renal function, represented by plasma creatinine concentration. Lastly, adjusted model 4 included the variables from model 3 and indicators of disease severity at admission, such as oxygen saturation, NT-proBNP, interleukin-6, ferritin, CRP, and procalcitonin. Multivariable models for these analyses were constructed using variables associated with in-hospital mortality at a significance level of *p* < 0.2.

To assess the association between myocardial injury and long-term all-cause mortality in discharged COVID-19 patients, we conducted a Cox multivariable regression analysis. Model 1 was calibrated for age and sex. Model 2 included the variables from model 1, and additional adjustments were made for comorbidities such as hypertension, coronary heart disease, atrial fibrillation, heart failure, diabetes, and chronic kidney disease. Model 3 further incorporated the variables from model 2, with the addition of renal function represented by plasma creatinine concentration. The selection of variables for these multivariable models was based on their association with long-term mortality at a significance level of *p* < 0.2. The construction of a Kaplan–Meier curve facilitated the visualization of the probability of survival over time, stratified by the presence or absence of myocardial injury.

Statistical significance was determined based on a two-sided *p*-value threshold of less than 0.05, indicating a level of confidence in the results.

## Results

**Figure 1:**
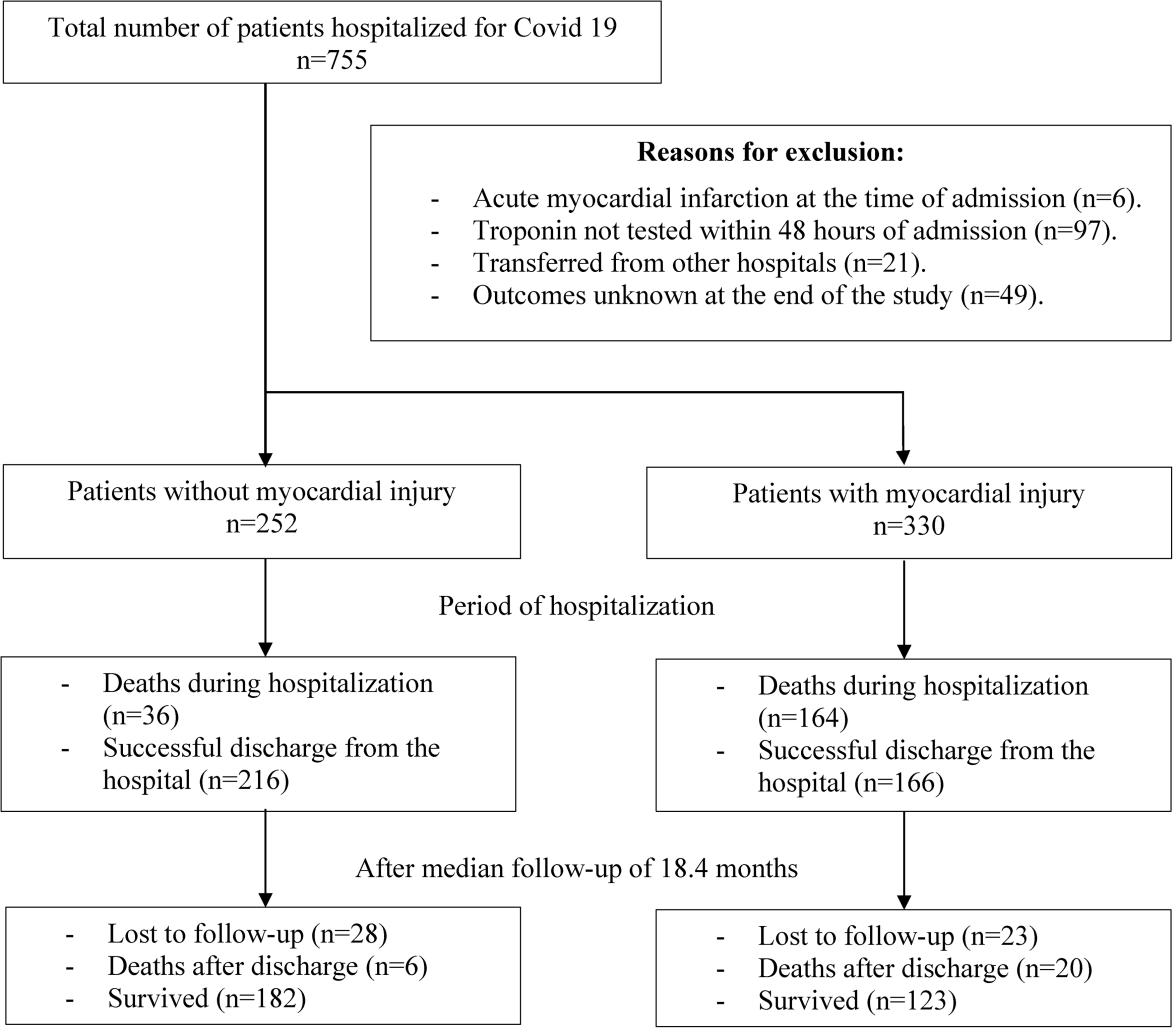
Flowchart of patients included in the study.

During the study period, a total of 755 patients with COVID-19 received treatment at UCICC. Of these, 706 patients were discharged at the end of the study. However, 124 cases were excluded from the study, which comprised 6 cases diagnosed with acute myocardial infarction at the time of admission, 21 cases transferred from other hospitals, and 97 cases that were not tested for serum troponin level within 48 h of admission. After excluding these cases, a total of 582 study subjects were included in the analysis (Figure 1).

### Study population characteristics

The study population had a mean age of 63.3 ± 16.2, with women accounting for 55.3% of the participants. The most prevalent comorbidities observed were hypertension (58.4%), chronic ischemic heart disease (20.4%), diabetes mellitus (38.8%), and chronic kidney disease (9.6%). The frequency of cardiovascular diseases was higher in patients with myocardial injury compared to those without myocardial injury. Table 1 shows the baseline characteristics of patients categorized based on the occurrence of myocardial injury.

**Table 1:** Baseline characteristics of patients with COVID-19.

Variables	Myocardial injury			
	All (*n* = 582)	Without (*n* = 252)	With (*n* = 330)	*p*-value
Age, year	63.3 (16.2)	57.3 (15.3)	67.9 (15.3)	<0.001
Female, no. (%)	322 (55.3)	134 (53.2)	188 (57.0)	0.400
Chronic medical conditions				
Hypertension, no. (%)	340 (58.4)	127 (50.4)	213 (64.5)	0.001
Coronary artery disease, no. (%)	119 (20.4)	37 (14.7)	82 (24.8)	0.003
Atrial fibrillation, no. (%)	30 (5.2)	4 (1.6)	26 (10.3)	<0.001
Heart failure, no. (%)	27 (4.6)	4 (1.6)	23 (7.0)	0.002
Diabetes mellitus, no. (%)	226 (38.8)	80 (31.7)	146 (44.2)	0.002
Chronic renal failure, no. (%)	56 (9.6)	7 (2.8)	49 (14.8)	<0.001
Cancer, no. (%)	18 (3.1)	6 (2.4)	12 (3.6)	0.386
COPD, no. (%)	40 (6.9)	16 (6.3)	24 (7.3)	0.663
General condition of patient on admission				
BMI, kg/m^2^	23.6 (3,9)	23.9 (7.7)	23.9 (4.0)	0.059
Heart rate, bpm	95.8 (20.0)	91.1 (17.2)	99.3 (21.3)	<0.001
SBP, mmHg	126.4 (23.2)	127.1 (19.3)	126.0 (25,8)	0.403
Oxygen saturation, %	89.4 (10.5)	92.1 (6.6)	87.4 (12.3)	<0.001
P/F ratio	172.2 (112.3)	189.0 (117.6)	160.1 (107.9)	0.003
Time of disease, day, median (IQR)	4.0 (2.0; 8.5)	5.0 (2.5; 11.5)	4.0 (2.0; 7.0)	0.114
Pregnancy, no. (%)	9 (1.5)	6 (2.4)	3 (1.0)	0.185

COPD, chronic obstructive pulmonary disease; BMI, body mass index; SBP, systolic blood pressure; P/F ratio, the ratio of arterial oxygen partial pressure (PaO2 in mmHg) to fractional inspired oxygen (FiO2).

Upon admission, the mean heart rate was 95.8 ± 20.0, mean peripheral blood oxygen saturation was 89.4 ± 10.5%, and the mean P/F ratio was 172.2 ± 112.3. High-sensitivity troponin T level was tested in 342 patients (58.8%), with a median value of 17.3 (interquartile range: 10.3–36.8) ng/L, while 265 patients (41.2%) underwent testing for high-sensitivity troponin I level, with a median value of 17.8 (interquartile range: 7.1–103.0) ng/L. Patients with myocardial injury have more abnormalities on tests of inflammation, kidney function (see Table 2). Table 3 provides details of the treatment administered to hospitalized COVID-19 patients. Compared to patients without myocardial injury, those with myocardial injury required more intensive care, including mechanical ventilation, vasopressors and antibiotics. The median hospital stay for all patients was 13.0 days, with an interquartile range of 9.0–20.0 days.

**Table 2:** Laboratory findings at admission of patients with COVID-19.

		Myocardial injury		*p*-value
	All (*n* = 582)	Without (*n* = 252)	With (*n* = 330)	
hs-cTn T, ng/L (*n* = 342)	17.3 (10.3–36.8)	9.5 (7.5–11.9)	31.5 (19.7–60.7)	
hs-cTn I, ng/L (*n* = 265)	17.8 (7.1–103.0)	6.9 (4.7–9.3)	83.2 (29.2–353.9)	
NT-proBNP, ng/L (*n* = 333)	354.0 (125.5–1,045.5)	141.0 (70.0–340.0)	601.0 (239.5–1,532.75)	<0.001
BNP, ng/L (*n* = 44)	149.5 (94.5–340.0)	95.0 (78.0–149.0)	196.5 (116.3–494.0)	0.005
WBC, G/L	9.8 (6.9–13.9)	9.0 (6.4–12.5)	10.5 (7.5–16.3)	<0.001
HGB, g/L	129.2 (19.9)	132.7 (18.0)	126.5 (20.8)	0.019
PLT, G/L	261.0 (115.0)	276.1 (115.2)	249.4 (113.6)	0.006
CRP, mg/L	56.9 (26.0–117.4)	42.7 (19.0–91.6)	68.9 (30.4–139.0)	<0.001
Procalcitonin, ng/ml	0.2 (0.1–0.8)	0.1 (0.1–0.3)	0.4 (0.1–1.2)	<0.001
IL-6, pg/ml	25.7 (10.5–76.0)	16.0 (7.5–35.8)	37.4 (15.4–113.7)	<0.001
Ferritin, ng/ml	1,094.8 (672.6–1,675.6)	994.2 (650.1–1,675.0)	1,173.4 (675.5–1,742.0)	0.126
D-Dimer, ng/ml	1,200 (669.0–2,896.0)	836.0 (509.5–1,433.3)	1,814.5 (925.3–4,476.5)	<0.001
Albumin, g/L	27.9 (4.7)	29.1 (4.6)	27.3 (4.6)	0.002
Glucose, mg/dl	175.0 (133.0–265.0)	163.0 (128.0–263.5)	186.0 (133.0–268.5)	0.170
Creatinine, mg/dl	0.87 (0.69–1.17)	0.8 (0.6–1.0)	1.0 (1.8–1.4)	<0.001
AST, U/L	52.0 (33.0–82.0)	46.0 (32.0–75.0)	57.0 (33.0–91.5)	0.029
ALT, U/L	44.0 (26.0–76.8)	48.0 (28.0–91.5)	41.0 (25.0–68.0)	0.022
Fibrinogen, g/L	5.1 (4.17–6.03)	5.2 (4.2–6.1)	5.0 (4.1–6.0)	0.138
HbA1C, %	6.7 (6.1–8.2)	6.5 (6.0–8.0)	6.8 (6.2–8.3)	0.139

hs-cTn T, high-sensitivity cardiac troponin T; hs-cTn I, high-sensitivity cardiac troponin I; NT-proBNP, N-terminal prohormone of brain natriuretic peptide; WBC, white blood cells; HGB, hemoglobin; PLT, platelet count; CRP C-reactive protein; IL-6, interleukin 6; AST, aspartate aminotransferase; ALT, alanine transaminase; HbA1C, hemoglobin A1c.

**Table 3:** Treatment and outcome of patients with COVID-19.

		Myocardial injury		*p*-value
	All (*n* = 582)	Without (*n* = 252)	With (*n* = 330)	
Admission respiratory status				
Breathing room air, no. (%)	34 (5.8)	16 (6.3)	18 (5.5)	<0.001
Nasal cannula, no. (%)	113 (19.4)	62 (24.6)	51 (15.5)	
Mask, no. (%)	83 (14.3)	52 (20.6)	31 (9.4)	
HFNC, no. (%)	229 (39.3)	104 (41.3)	125 (37.9)	
NIV, no. (%)	9 (1.5)	3 (1.2)	6 (1.8)	
Invasive mechanical ventilation, no. (%)	114 (19.6)	15 (6.0)	99 (30.0)	
Vasopressor agents, no. (%)	29 (5.0)	4 (1.6)	25 (7.6)	0.001
Pulse steroid, no. (%)	61 (10.5)	30 (11.9)	31 (9.4)	0.327
Tocilizumab, no. (%)	3 (0.5)	2 (0.8)	1 (0.3)	0.413
Remdesivir, no. (%)	317 (54.5)	151 (59.9)	166 (50.3)	0.021
Antibiotic treatment, no. (%)	560 (96.2)	236 (93.7)	324 (98.2)	0.005
Anticoagulation, no. (%)	549 (94.3)	242 (96.0)	307 (93.0)	0.121
Duration of treatment, day	13 (9; 20)	13 (10; 20)	13 (8; 20)	0,413
Death, *n* (%), no. (%)	200 (34.4)	36 (14.3)	164 (49.7)	<0.001

HFNC, high-flow nasal cannula; NIV, non-invasive ventilation.

### Myocardial injury in patients with COVID-19

Our study found that 330 out of 582 patients (56.8%) with SARS-CoV-2 infection had myocardial injury upon admission. Multivariable logistic regression analysis revealed that age (OR = 1.038, 95% CI 1.020–1.057, *p* < 0.001), saturation of peripheral oxygen (OR = 0.964, 95% CI 0.940–0.988, *p* = 0.004), interleukin-6 (log interleukin-6, OR = 1.662, 95% CI 1.111–2.485, *p* = 0.013) and creatinine (OR = 3.304, 95% CI 1.705–6.404, *p* < 0.001) were independent risk factors for myocardial injury in these patients (Supplementary Table S1).

### Myocardial injury as an independent risk factor for in-hospital mortality

Among 582 hospitalized patients, 200 deaths were recorded, accounting for 34.4% of all causes. Univariate analysis of factors associated with in-hospital mortality can be found in Table 4. The presence of myocardial injury was associated with a significant increase in in-hospital mortality among patients hospitalized with COVID-19 (OR = 5.928, 95% CI 3.920–8.964, *p* < 0.001). This association remained statistically significant even after adjusting for demographic factors (OR = 4.871, 95% CI 3.174–7.475, *p* < 0.001, model 1), comorbidities (OR = 4.885, 95% CI 3.161–7.547, *p* < 0.001, model 2), renal function (OR = 4.334, 95% CI 2.796–6.717, *p* < 0.001, model 3), and severity at admission (OR = 3.758, 95% CI 1.854–7.678, *p* < 0.001, model 4) (see Table 5).

**Table 4:** Univariate analysis of factors associated with in-hospital mortality in patients hospitalized with COVID-19.

Variables	OR	95% CI	*p*-value
Myocardial injury	5.928	3.920–8.964	<0.001
Age, year	1.036	1.024–1.049	<0.001
Female	1.119	0.794–1.578	0.521
Hypertension	1.472	1.035–2.096	0.032
Coronary artery disease	1.383	0.914–2.094	0.125
Atrial fibrillation	1.493	0.710–3.139	0.291
Heart failure	2.136	0.984–4.638	0.055
Diabetes mellitus	1.847	1.303–2.619	0.001
Chronic renal failure	1.900	1.091–3.309	0.023
Cancer	1.550	0.602–3.991	0.364
COPD	1.297	0.672–2.502	0.438
SBP	0.995	0.988–1.003	0.230
SpO2	0.948	0.932–0.965	<0.001
Log NT-proBNP	2.119	1.478–3.038	<0.001
WBC	1.082	1.052–1.112	<0.001
HGB	0.998	0.990–1.007	0.697
PLT	0.997	0.995–0.998	<0.001
CRP	1.007	1.004–1.009	<0.001
Procalcitonin	1.166	1.083–1.255	<0.001
Log IL-6	3.712	2.629–5.240	<0.001
Log Ferritin	2.931	1.739–4.942	<0.001
Log D-Dimer	0.778	0.561–1.079	0.132
Albumin	0.929	0.886–0.973	0.002
Glucose	1.002	1.001–1.004	0.001
Creatinine	1.817	1.354–2.438	<0.001
AST	1.000	0.999–1.002	0.825
ALT	0.998	0.996–1.000	0.105
Fibrinogen	0.992	0.882–1.116	0.895

COPD, chronic obstructive pulmonary disease; BMI, body mass index; SBP, systolic blood pressure; P/F ratio, the ratio of arterial oxygen partial pressure (PaO2 in mmHg) to fractional inspired oxygen (FiO2); hs-cTn T, high-sensitivity cardiac troponin T; hs-cTn I, high-sensitivity cardiac troponin I; NT Pro BNP, N-terminal prohormone of brain natriuretic peptide; WBC, white blood cells; HGB, hemoglobin; PLT, platelet count; CRP C-reactive protein; IL-6, interleukin 6; AST, aspartate aminotransferase; ALT, alanine transaminase.

**Table 5:** Odds ratio of the association between myocardial injury and in-hospital mortality in COVID-19 patients.

	OR	95% CI	*p*-value
Unadjusted	5.928	3.920–8.964	<0.001
Model 1	4.871	3.174–7.475	<0.001
Model 2	4.885	3.161–7.547	<0.001
Model 3	4.334	2.796–6.717	<0.001
Model 4	3.758	1.854–7.678	<0.001

Model 1 was adjusted for age, sex, and myocardial injury. Model 2 was adjusted to include the variables from model 1, along with comorbidities such as hypertension, coronary heart disease, atrial fibrillation, heart failure, diabetes, and chronic kidney disease. Model 3 expanded upon the variables in model 2 by incorporating kidney function. Model 4 included the variables from model 3 and additional variables related to disease severity, namely oxygen saturation, CRP, procalcitonin, interleukin-6, and ferritin. OR, odds ratio; CI, confidence interval; CRP, C-reactive protein.

### Long-term prognosis of hospitalized COVID-19 patients with myocardial injury

**Figure 2:**
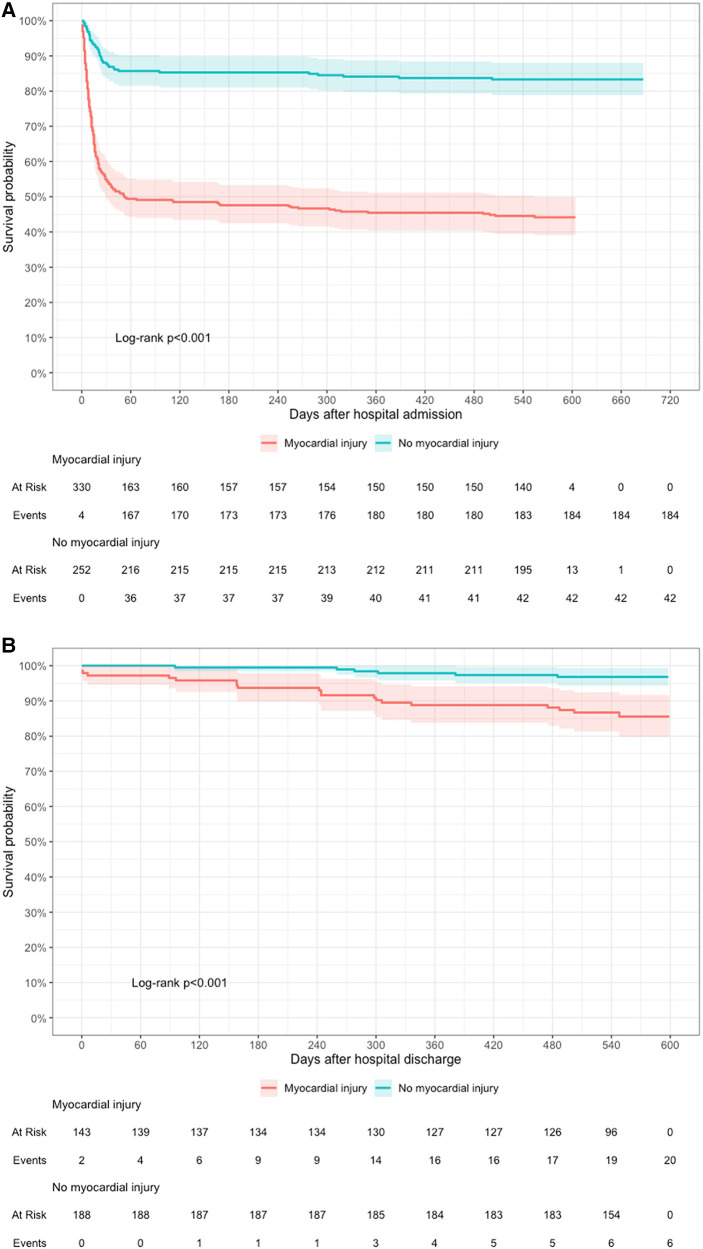
Relationship between myocardial injury and long-term all-cause mortality in hospitalized COVID-19 patients. (**A**) Kaplan–Meier survival curves illustrating the probability of survival with and without myocardial injury at the time of admission. (**B**) Kaplan–Meier survival curve illustrating the probability of survival with and without myocardial injury after hospital discharge.

A total of 382 patients were successfully discharged following hospitalization for COVID-19. Among them, 331 patients (86.6%) achieved a documented survival outcome, with a median follow-up time of 18.4 months (IQR 18.0–18.8). However, 51 patients were lost to follow-up, accounting for 13.4% of the cohort. During the follow-up period, 26 all-cause deaths were recorded (7.9%). Comparing the mortality rates, the group with myocardial injury had a significantly higher rate of 14.0% (20/143), while the group without myocardial injury had a rate of 3.2% (6/188) (*p* < 0.001). Univariate analysis revealed that myocardial injury was associated with an increased risk of long-term mortality in successfully discharged COVID-19 patients discharged (HR = 4.684, 95% CI 1.881–11.664, *p* = 0.001) (Table 6). However, after adjusting for demographic factors (HR = 2.022, 95% CI 0.766–5.338, *p* = 0.155, model 1), comorbidities (HR = 2.143, 95% CI 0.805–5.704, *p* = 0.127, model 2), and renal function (HR = 2.128, 95% CI 0.792–5.712, *p* = 0.134, model 3), this association was no longer statistically significant (Table 7; Figure 2).

**Table 6:** Univariate analysis of factors associated with long-term mortality in discharged COVID-19 patients.

Variables	HR	95% CI	*p*-value
Myocardial injury	4.684	1.881–11.664	0.001
Age	1.097	1.065–1.129	<0.001
Female	0.764	0.347–1.683	0.504
Hypertension	1.812	0.807–4.065	0.149
Coronary artery disease	1.072	0.404–2.843	0.889
Atrial fibrillation	4.520	1.557–13.125	0.006
Heart failure	7.215	2.714–19.175	<0.001
Diabetes mellitus	1.264	0.574–2.786	0.561
Creatinine	1.068	0.774–1.473	0.691

**Table 7:** Hazard ratio of the association between myocardial injury and long-term mortality in discharged COVID-19 patients.

	HR	95% CI	*p*-value
Unadjusted	4.684	1.881–11.664	0.001
Model 1	2.022	0.766–5.338	0.155
Model 2	2.143	0.805–5.704	0.127
Model 3	2.128	0.792–5.712	0.134

Model 1 was adjusted for age, sex, and myocardial injury. Model 2 included the variables from model 1 and further adjusted for comorbidities, including hypertension, coronary heart disease, atrial fibrillation, heart failure, diabetes, and chronic kidney disease. Model 3 incorporated the variables from model 2 and additionally adjusted for kidney function. HR, hazard ratio; CI, confidence interval.

## Discussion

Our study provided valuable information on myocardial injury in COVID-19 hospitalized patients. We observed that 56.8% of patients exhibited myocardial injury, which was associated with a 3.8-fold increase in the risk of in-hospital mortality compared to those without myocardial injury. After a median follow-up period of 18.4 months, we determined an overall all-cause mortality rate of 7.9% among successfully discharged COVID-19 patients. Our findings did not reveal a significant association between myocardial injury and long-term all-cause mortality.

### Myocardial injury in hospitalized COVID-19 patients

The impact of SARS-CoV-2 on the cardiovascular system is diverse and can manifest in various ways, such as type 1 or type 2 acute myocardial infarction, arterial or venous thrombosis, acute myocarditis-pericarditis, arrhythmia, acute heart failure, cardiogenic shock, or cardiac arrest. Myocardial injury, as defined by elevated cardiac enzymes >99th percentile, is the most frequently observed presentation among these manifestations (15). The frequency of acute myocardial injury in COVID-19 patients is currently unknown and varies significantly between studies (ranging from approximately 7%–36%), due to differences in study population characteristics, diagnostic criteria, reference value thresholds, and sampling time for troponin (1, 5–7). A meta-analysis of 57 studies involving 34,072 hospitalized COVID-19 patients found that the incidence rate of acute myocardial injury was 21%, with the severity group alone being 50%, which is higher than that observed in other respiratory viruses (16). Notably, our study reported an even higher incidence rate of acute myocardial injury at admission, with a value of 56.8%, exceeding many previous reports. There was no difference in the sex ratio between groups with and without myocardial injury, which contrasts with some previous analyses suggesting that men are more susceptible to developing myocardial injury and experiencing severe outcomes such as acute respiratory failure, invasive mechanical ventilation, and ICU admission (17). Patients with concomitant cardiovascular diseases, such as hypertension (58.4%), chronic coronary artery disease (20.4%), and atrial fibrillation (5.2%), had a statistically significant higher incidence rate of myocardial injury compared to patients without comorbid cardiovascular disease. This is likely due to an increase in ACE2 receptor expression, making it easier for the virus to directly attack cardiac muscle cells. Additionally, most patients were hospitalized with severe and critical conditions, resulting in a high rate of invasive mechanical ventilation (19.6%) and vasopressor use (5%). Most studies have shown that the incidence of acute myocardial injury increases with disease severity (16).

Diagnostic criteria also cause differences in the incidence of acute myocardial injury between different reports. Most studies rely solely on the elevation of cardiac enzymes due to the pandemic context and difficulty in accessing other diagnostic facilities such as electrocardiogram, echocardiography, cardiac magnetic resonance, and myocardial biopsies. However, while biomarkers are highly sensitive, they are not specific for myocardial injury (12). There are many theories about the cause of elevated cardiac enzymes, but the specific cause in each patient is often unknown (18). Nonetheless, troponin remains a widely accepted biomarker and is an important part of the initial evaluation in patients hospitalized for COVID-19.

Our study revealed that patients with acute myocardial injury had more severe acute illness and showed more abnormalities in their blood test results, such as elevated levels of CRP, procalcitonin, IL-6, ferritin, D-Dimer, and creatinine, as well as decreased levels of albumin. These patients also required more mechanical ventilation, which is consistent with previous findings. Therefore, acute myocardial injury likely reflects an ongoing pathology due to cytokine release and tissue hypoxia, indicating a strong correlation between acute myocardial injury and poor outcomes in hospitalized COVID-19 patients (5, 6, 9, 19).

### Myocardial injury as an independent risk factor for in-hospital mortality

Evaluation of lung function is a key determinant in the diagnosis, stratification, treatment, and prognosis of patients with SARS-CoV-2 infection (20). A meta-analysis revealed that myocardial injury was associated with a significant 10.6-fold increase in adverse events, encompassing outcomes such as death, clinical severity, the requirement for ICU treatment, and mechanical ventilation (19). In a study of 2,736 COVID-19 patients in New York, the overall mortality rate was 18.5%, and even a slight increase in hs cTnI (0.03–0.09 ng/ml) was associated with a higher risk of death (HR 1.75, 95% CI 1.37–2.24) (7). In our study, we observed an in-hospital all-cause mortality rate of 34.4%. After adjusting for age, gender, comorbidities, renal function, and disease severity at hospital admission, myocardial injury emerged as a significant independent risk factor, increasing the risk of in-hospital mortality by 3.8 times compared to patients without myocardial injury. Patients with myocardial injury are often older and have a history of cardiovascular disease, and use ACE inhibitors or angiotensin receptor blockers. Data from 44,672 patients with COVID-19 from the Chinese Center for Disease Control and Prevention demonstrated that patients with cardiovascular disease had a higher mortality rate (10.5% vs. 0.9%) (21). Care should be taken when interpreting the results of multivariate analysis because the model may not be robust enough to evaluate the independent relationship of hs cTn and other predictors. Therefore, it remains uncertain whether acute myocardial injury is an associated manifestation or an independent risk factor for adverse events.

### Long-term prognosis of hospitalized COVID-19 patients with myocardial injury

There was a paucity of data regarding the long-term prognosis of COVID-19 patients with myocardial injury. Understandably, during a pandemic, the primary focus was on early diagnosis and treatment to minimize complications arising from systemic inflammation and prevent multi-organ failure. However, as the number of recovered COVID-19 patients increased and concerns about potential long-term cardiovascular consequences emerged, it became crucial to identify predictors that could aid in selecting patients who would benefit from intensive monitoring and treatment. This approach also ensured the appropriate and cost-effective allocation of medical resources (22). Nonetheless, only a limited number of studies have investigated the role of myocardial injury in patients who survive hospitalization for COVID-19 (5, 11, 23–25). Some studies have demonstrated a significant association between myocardial injury and adverse long-term outcomes, particularly increased readmissions (10, 24). It is hypothesized that cardiac injury occurring during the acute phase persists after hospital discharge due to an ongoing inflammatory response, along with changes in cardiac morphology and function such as myocardial fibrosis or edema. This persistence may result in a higher risk of heart failure, arrhythmias, and mortality (24). Recent evidence supports this hypothesis, showing that 60% of recovered COVID-19 patients exhibited signs of myocarditis on cardiac magnetic resonance imaging (26). To the best of our knowledge, this study had the longest follow-up period (median 18.4 months). Since COVID-19 is primarily considered an acute illness, most patients are not routinely scheduled for post-discharge follow-up and examinations. Consequently, there are no specific guidelines for the long-term management of COVID-19 patients, leading to challenges in collecting data on patient outcomes after hospital discharge. However, through our efforts, we successfully obtained vital information from 86.6% of discharged COVID-19 patients. The all-cause mortality rate during the follow-up period was found to be 7.9%. Our study identified a statistically significant difference in all-cause mortality between patients with and without myocardial injury. Nevertheless, after adjusting for age, sex, comorbid cardiovascular disease, and renal function, myocardial injury was not found to be a predictor of long-term mortality in successfully discharged COVID-19 patients. This finding is consistent with a prior study involving 392 discharged COVID-19 patients, which showed that the incremental mortality over 12 months remained low, even among patients with troponin positivity (25). These results provide a new perspective on the importance of cardiac injury in COVID-19 patients. While myocardial injury during the acute phase strongly correlates with in-hospital mortality, it does not appear to be associated with long-term mortality after successful discharge from the hospital. However, additional research is necessary to ascertain if this trend continues during longer-term follow-up and to investigate other potential impacts, such as re-hospitalization and post-COVID symptoms. Future studies will contribute to a more comprehensive understanding of these factors.

### Limitations

Limitations of our study should be noted. Firstly, due to the retrospective study design, there were instances where patients did not provide complete information or were lost to follow-up, which may have introduced potential bias. Secondly, the diagnosis of myocardial injury relied solely on elevated troponin levels and did not incorporate further assessments such as electrocardiogram, echocardiogram, or cardiac magnetic resonance imaging. Thirdly, our study only included hospitalized patients at a single center, most of whom had severe or critical disease, therefore findings may not be generalizable to the broader population infected with SARS-CoV-2. Fourthly, troponin kinetics were not continuously monitored. Lastly, our assessment of long-term prognosis in patients with SARS-CoV-2 infection with myocardial injury only focused on all-cause mortality, overlooking other important outcomes such as re-hospitalization, thromboembolic complications, arrhythmias, myocardial infarction and heart failure.

## Conclusion

Myocardial injury is a prevalent complication and exhibits a strong association with in-hospital mortality in COVID-19 hospitalized patients. However, our study reveals that in successfully discharged COVID-19 patients, myocardial injury during the acute phase does not predict long-term all-cause mortality. These results imply that a less aggressive long-term management approach may be considered for this patient group, leading to cost reduction and more appropriate allocation of medical resources. It is important to note that further data collection over an extended follow-up period is needed to monitor any potential changes in this trend.

## Data Availability

The original contributions presented in the study are included in the article/**Supplementary Material**, further inquiries can be directed to the corresponding author.
