# Supplementary material for: Prevalence and impact of myocardial injury among patients hospitalized with COVID-19
Source: Front Cardiovasc Med. 2023 Aug 1;10:1202332. doi: 10.3389/fcvm.2023.1202332 (PMC10433191; doi:10.3389/fcvm.2023.1202332)
Supplement: Supplementary file 1 [file Datasheet1.docx]

Supplementary Material

**Prevalence and Impact of Myocardial Injury among Patients Hospitalized with COVID-19**

Vu Hoang Vu^1,2^, Thanh Cong Nguyen^2*^, Quang Dang Duy Pham2, Dan Ngoc Pham3, Le Bao Le4, Khoi Minh Le5

^1^Faculty of Medicine, University of Medicine and Pharmacy at Ho Chi Minh City, Ho Chi Minh City, Vietnam.

^2^Interventional Cardiology Department, University Medical Center Ho Chi Minh City, Ho Chi Minh City, Vietnam.

^3^Department of Cardiology, University Medical Center Ho Chi Minh City, Ho Chi Minh City, Vietnam.

^4^Rheumatology Department, University Medical Center Ho Chi Minh City, Ho Chi Minh City, Vietnam.

^5^Cardiac Imaging Unit, University Medical Center Ho Chi Minh City, Ho Chi Minh City, Vietnam.

*** Correspondence:**Thanh Cong Nguyen
[thanh.nc@umc.edu.vn](mailto:thanh.nc@umc.edu.vn)

**Methods S1. Definition of severity grades of COVID-19 patients based on the Guidelines for Diagnosis and Treatment of COVID-19 issued by the Ministry of Health of Vietnam (2021)**

1. **Mild severity**

- Patients with COVID-19 exhibit non-specific clinical symptoms such as fever, dry cough, sore throat, nasal congestion, fatigue, headache, muscle aches, loss of taste, smell, and diarrhea.
- Breathing rate is below 20 breaths per minute, and oxygen saturation (SpO2) is above 96% when breathing ambient air.
- The patient is awake and capable of self-care.
- Chest X-ray shows either normal findings or minimal damage.

1. **Moderate severity**
   1. Clinical

- General condition: The patient experiences mild severity with non-specific clinical symptoms.
- Respiratory: Signs of pneumonia are present, with dyspnea, rapid breathing at a rate of 20-25 breaths per minute, lung crackles, and no indications of severe respiratory failure. SpO2 ranges from 94% to 96% when breathing ambient air. The patient may experience difficulty breathing during exertion, such as walking around the house or climbing stairs.
- Circulation: The pulse may be fast or slow, with dry skin, tachycardia, and normal blood pressure. The patient is conscious and alert.
  1. Subclinical
- Chest X-ray and chest CT reveal lesions covering less than 50% of the lung area.
- Ultrasound shows B-lines images.
- Arterial blood gas analysis indicates a PaO2/FiO2 ratio greater than 300.

1. **Severe Severity**
   1. Clinical

- Respiratory: Signs of pneumonia are present along with any of the following: respiratory rate exceeding 25 breaths per minute, severe shortness of breath, use of accessory respiratory muscles, or SpO2 below 94% when breathing ambient air.
- Circulation: Tachycardia or possibly bradycardia may occur, with normal or elevated blood pressure.
- Nervous: The patient may exhibit restlessness, lethargy, or fatigue.
  1. Subclinical
- Chest X-ray and chest CT reveal lesions covering more than 50% of the lung area.
- Arterial blood gas analysis indicates a PaO2/FiO2 ratio between 200 and 300.
- Ultrasound shows multiple B-lines.

1. **Critical Severity**
   1. Clinical

- Respiratory: The patient exhibits rapid breathing exceeding 30 breaths per minute or less than 10 breaths per minute, with signs of severe respiratory failure characterized by labored breathing and abnormal patterns.
- Nervous: The patient experiences decreased consciousness or falls into a coma.
- Circulation: Tachycardia, possibly bradycardia, and low blood pressure are observed.
- Kidney: There is a decrease in urine output or anuria.
  1. Subclinical
- Chest X-ray and chest CT reveal lesions covering more than 50% of the lung area.
- Arterial blood gas analysis indicates a PaO2/FiO2 ratio below 200, respiratory acidosis, and blood lactate levels exceeding 2 mmol/L.
- Ultrasound shows multiple B-lines images.

**Methods S2. Inclusion and Exclusion Criteria**

1. **Inclusion criteria**

All of the following criteria must be met:

- The patient was diagnosed with COVID-19 based on a positive real-time polymerase chain reaction (RT-PCR) test for SARS-CoV-2, in accordance with the diagnostic and treatment guidelines issued by the Ministry of Health of Vietnam. Clinical symptoms were also taken into consideration for the diagnosis
- The patient underwent at least one high-sensitivity cardiac troponin test (hs-cTn) within 48 hours of admission, regardless of whether troponin I or troponin T was used.

1. **Exclusion criteria**

The following criteria were used to exclude patients from the study

- Age less than 18 years.
- The patient was diagnosed with acute coronary syndrome, including acute ST-segment elevation myocardial infarction or non-ST-segment elevation myocardial infarction, according to the Fourth Universal Definition of Myocardial Infarction (2018).
- The patient was referred from another disease with similar functionality and classification.

**Table S1. Independent risk factors for myocardial injury in patients hospitalized with COVID-19.**

| **Variables** | **OR** | **95%CI** | **p-value** |
| --- | --- | --- | --- |
| Age | 1.038 | 1.020 – 1.057 | <0.001 |
| Hypertension | 0.885 | 0.531 – 1.477 | 0.641 |
| Coronary artery disease | 1.379 | 0.739 – 2.570 | 0.312 |
| Atrial fibrillation | 2.520 | 0.727 – 8.736 | 0.145 |
| Heart failure | 2.049 | 0.551 – 7.624 | 0.284 |
| Diabetes mellitus | 1.078 | 0.665 – 1.748 | 0.760 |
| Chronic renal failure | 1.435 | 0.499 – 4.127 | 0.502 |
| Oxygen saturation | 0.964 | 0.940 – 0.988 | 0.004 |
| White blood cell | 1.103 | 1.053-1.155 | <0.001 |
| C-reactive protein | 1.003 | 0.999 – 1.007 | 0.113 |
| Procalcitonin | 1.021 | 0.954 – 1.092 | 0.550 |
| log Interleukin-6 | 1.662 | 1.111-2.485 | 0.013 |
| log D-Dimer | 1.117 | 1.784 – 1.669 | 0.588 |
| Creatinine | 3.304 | 1.705-6.404 | <0.001 |

OR, odds ratio; CI, confidence interval.
